# Effects of cinnamon supplementation combined with exercise training on anthropometric indices and reproductive hormones in overweight and obese women

**DOI:** 10.1080/15502783.2026.2724102

**Published:** 2026-09-02

**Authors:** Fatemeh Ahmadi, Mohammad Mehdi Khaleghi, Abdossaleh Zar

**Affiliations:** a Department of Sport Science, School of Literature and Humanities, Persian Gulf University, Bushehr, Iran; b Persian Gulf Sports, Nutrition and Wellness Research and Technology Group, School of Literature and Humanities, Persian Gulf University, Bushehr, Iran; c Department of Exercise Physiology, Faculty of Sport Sciences, Shahid Chamran University of Ahvaz, Ahvaz, Iran

**Keywords:** Exercise, cinnamon, abdomen circumference, chest circumference, sex hormones, obesity

## Abstract

**Background:**

The prevalence of obesity and increased fat in the chest and abdomen in women and hormonal changes, can affect female fertility. The present study aims to investigate the effect of exercise training combined with cinnamon supplementation on chest and abdominal circumference, as well as sex hormones, in overweight and obese women.

**Methods:**

Twenty-six inactive overweight and obese women were randomly allocated to an exercise (E; *n* = 13) and exercise plus cinnamon supplementation (E+S; *n* = 13) group. Both groups completed a 4-week exercise program three times weekly, while the E+S group additionally received cinnamon supplementation three times weekly. Chest and abdominal circumferences and serum concentrations of estradiol, progesterone, follicle-stimulating hormone (FSH), and luteinizing hormone (LH) were assessed before and after the intervention.

**Results:**

Significant pre-to-post reductions in chest and abdominal circumference were observed in both groups (all *p* ≤ 0.005). Estradiol, progesterone, and FSH concentrations increased, whereas LH concentrations decreased significantly in both groups (all *p* ≤ 0.005). However, between-group comparisons revealed a significant difference only for chest circumference (*p* = 0.003), with no significant group differences in abdominal circumference or any reproductive hormone concentration (all *p* > 0.05).

**Conclusion:**

Both groups showed favorable changes in anthropometric and reproductive hormone measures following the 4-week study period. However, the significant between-group difference was limited to chest circumference, providing no clear evidence that cinnamon supplementation conferred additional benefits for abdominal circumference or reproductive hormone profiles beyond those observed with exercise. These findings highlight the potential relevance of exercise-based interventions while warranting confirmation in larger, adequately powered trials.

## 1. Introduction

Obesity and fertility in women of reproductive age represent a complex and increasingly relevant topic in recent research. Obesity is not merely an excess of body weight; rather, it is a systemic disease that significantly impacts hormonal balance, metabolic function, and overall health [1]. The American Medical Association recognizes obesity as a disease and highlights its extensive effects on health, including female reproductive health [2]. Studies have demonstrated that obesity is a chronic, multifactorial condition characterized by excessive adipose tissue accumulation and associated metabolic, biomechanical, and psychological disorders [2]. It can lead to hormonal imbalances, ovulatory dysfunction, reduced efficacy of infertility treatments, and increased pregnancy complications [3]. According to the guidelines of the World Health Organization (WHO) and the Centers for Disease Control and Prevention (CDC), a body mass index (BMI) between 25 and 30 kg/m^2^ is classified as overweight, a BMI of 30 or higher as obese, and a BMI of 40 or higher as severe obesity [1].

Studies have shown that in the United States, 41.9% of women are classified as obese, while 11.9% suffer from severe obesity, highlighting the high prevalence of this issue in modern societies [4]. In addition to increasing the risk of cardiovascular diseases, type 2 diabetes, and stroke, obesity has significant implications for fertility like pregnancy loss [3,5]. Overweight and obese women experience longer time-to-pregnancy and lower fertility success rates [3]. These individuals require higher doses of gonadotropins and have an increased risk of miscarriage [6,7]. Research has indicated that the underlying causes of these reproductive challenges include menstrual irregularities, reduced oocyte quality, and implantation failure due to endometrial abnormalities [3,8–10]. Notably, additional studies have demonstrated that overall obesity, particularly android (abdominal) obesity, plays a more critical role in the development of these disorders [10–13].

Therefore, weight management through lifestyle modifications like physical activity [14], dietary interventions, and pharmacological treatments has been shown to may have positive effects on fertility [15]. Studies indicate that even a modest amount of weight loss can lead to improved hormonal function, regulation of the menstrual cycle, and increased chances of conception [3]. Consequently, weight control is recommended not only for overall health but also as a crucial factor in enhancing fertility and reducing pregnancy complications [15].

In this regard, research has demonstrated that exercise can induce various changes in sex hormone levels, including reductions in serum follicle-stimulating hormone (FSH), luteinizing hormone (LH), estrogen, estradiol, total testosterone, and free testosterone [16–19]. Additionally, exercise has been shown to improve cortisol levels [19,20], increase salivary concentrations of testosterone and dehydroepiandrosterone (DHEA) [21], and enhance sex hormone-binding globulin (SHBG) levels [18]. These hormonal adaptations are of particular importance, as fluctuations in sex hormone levels—especially estradiol—have been linked to changes in body composition, including reductions in fat mass, skeletal muscle mass, and overall fat-free mass [22]. Furthermore, variations in serum free testosterone, commonly expressed as the free androgen index, have been shown to correlate with alterations in fat-free mass [23]. In addition, exposure to testosterone has been associated with a leaner body composition—characterized by decreased body fat percentage and increased fat-free mass—in physically active young women [24]. Conversely, obesity has been shown to elevate cortisol concentrations in both serum and urine in women [25]. Therefore, careful monitoring and consideration of these hormonal parameters are essential when evaluating the physiological effects of exercise and body composition outcomes.

It has been demonstrated that spinning, resistance, and combined training (resistance plus spinning) significantly reduce serum levels of resistin and visfatin, as key adipocytokines secreted by adipose tissue that have a positive correlation with body mass [26], in overweight women, with combined training exerting greater effects than either modality alone [27]. Similarly, Lotfi et al. reported significant pre- to post-intervention change in resistin and visfatin following a combined exercise program in women with overweight status [28]. Furthermore, these interventions improved lipid profile indices, including reductions in total cholesterol, triglycerides, and LDL, alongside an increase in HDL, with the most pronounced benefits observed in the combined training group. Importantly, such programs also enhanced both physical and psychological dimensions of quality of life [27]. In another study, although yoga and aerobic training did not produce statistically significant effects on body weight, body fat percentage, HOMA-IR, or circulating irisin levels, the participants' increased motivation to engage in these activities—particularly yoga—suggests their potential as complementary exercise strategies for women with type 2 diabetes [29].

On the other hand, polyphenolic compounds, especially those derived from medicinal plants, have attracted attention for their potential to modulate metabolic and endocrine functions. Notably, these compounds can not only enhance the positive effects of physical activity but also mitigate some of the adverse responses induced by exercise [30–34]. Among these, cinnamon (Cinnamomum zeylanicum) has demonstrated multiple physiological and health benefits [35,36]. Cinnamon has traditionally been used to help regulate menstrual cycles in women with polycystic ovary syndrome (PCOS), a condition also described as polyendocrine metabolic ovarian syndrome (PMOS), which is frequently associated with obesity and insulin resistance [37]. In addition, cinnamon consumption by overweight inactive women has also been shown to improve their body composition, including weight, body fat percentage, and BMI [38]. Several studies have reported that cinnamon supplementation can reduce BMI, body weight [39], and serum testosterone levels in women with PCOS [40], suggesting its potential role in improving reproductive and metabolic health. However, in another study, although cinnamon extract supplementation at a dosage of 3 g per day for 12 consecutive weeks significantly reduced total testosterone levels in women with PCOS, it had no significant effects on their metabolic or hormonal indices [41]. Additionally, a study by Haji Manfared Nejad et al. (2018) found no significant changes in testosterone and dehydroepiandrosterone sulfate (DHEAS) levels following cinnamon supplementation [42].

Despite growing evidence supporting the individual benefits of physical activity and herbal supplementation, few studies have explored their combined effects on reproductive health, particularly in inactive overweight and obese women. This gap is significant given the complex interplay between metabolic status, hormonal regulation, and lifestyle interventions in this population. To address this, the present study investigates the synergistic effects of selected exercises and cinnamon supplementation, as an innovation, on body composition (waist and chest circumference) and key reproductive hormones (estradiol, progesterone, FSH, and LH) in overweight and obese women. Because the hypotheses of this study are that the synergy of selected exercises and cinnamon supplementation can affect waist and chest circumference and reproductive hormones (estradiol, progesterone, FSH, and LH) in overweight and obese women. The findings aim to provide valuable insights into non-pharmacological strategies for hormonal regulation, improving reproductive health, and managing obesity.

## 2. Methodology

### 2.1. Subjects

Before the start of the intervention, the sample size was determined using G*Power software (v. 3.1). The calculation assumed a moderate to large effect size (Cohen's f = 0.40), a significance level of 0.05, and 95% statistical power. Accounting for an anticipated 10% attrition, a total of 26 participants was deemed necessary to provide sufficient power to detect significant effects within the study.

The current study was conducted at the Department of Sport Sciences at Persian Gulf University (Bushehr, Iran). Twenty-six inactive female students with overweight or obesity (BMI ≥ 25) were randomly assigned to one of two groups: an exercise-only group (E*, n* = 13) and an exercise plus supplementation group (E+S, *n* = 13), using a computer-generated randomization procedure. Inclusion criteria included single individuals who were overweight or obese (BMI ≥ 25 kg/m^2^ and abdominal circumference ≥ 90 cm), had overall good health status, not using any specific medications, regularly consumed university-provided meals, and self-reported being in the luteal phase of the menstrual cycle at the time of baseline assessment. Participants were excluded if they had any medical disorders, were in the follicular phase of their menstrual cycle, had abnormal menstruation, or lived outside the university dormitory and did not consume meals provided by the university.

### 2.2. Implementation method

First, during the call in the student dormitory environment and explanation of the study goals and process, twenty-six inactive female students with overweight and obesity (BMI ≥ 25) who met the study entry criteria and agreed to participate in the study were selected and randomly placed in two exercise groups (E) (13 participants) and an exercise + supplement group (E + S) (13 participants). Then, the first stage of measuring the variables was performed. Subsequently, the subjects of both groups participated in their respective exercise programs for four weeks, and 24 hours after the last exercise session, the second stage of measuring the variables was performed.

### 2.3. Exercise training protocol

The exercise intervention was conducted for four weeks, with three supervised sessions per week, each lasting approximately 60 minutes [43]. Each training session consisted of a 10-minute warm-up (including stretching and flexibility exercises), a main exercise component, and a 10-minute cool-down period (including stretching and flexibility exercises).

The main exercise program comprised 16 bodyweight exercises, including dead bug, crunches, scissor crunches, straight-leg crunches, Russian twists, mountain climbers, reverse crunches, touch crunches, bicycle crunches, 90-degree leg crunches, leg curls, open-leg jumps with opposite-knee elbow raises, push-ups, wall push-ups, breaststroke movements with vertical jumps, and cross-arm planks. Exercises were performed three times per week in three sets of 12 repetitions, with a 30-second recovery period between sets. The training volume and structure remained constant throughout the intervention, and no progression in training load was implemented during the four-week period [44].

To ensure protocol compliance and consistency of exercise execution, all training sessions were conducted under the direct supervision of the investigator. Participant adherence was monitored throughout the intervention, and the investigator verified the completion of all prescribed exercises, sets, and repetitions while also overseeing exercise technique and movement execution. Exercise intensity, heart rate responses, ratings of perceived exertion, and total training load were not objectively assessed because of logistical limitations. This limitation should be considered when interpreting the physiological responses to the intervention. The selection of a four-week intervention period was based on previous studies examining the effects of exercise and nutritional supplementation on anthropometric and hormonal outcomes [45].

### 2.4. Supplementation

Participants assigned to the supplementation arm consumed 500 mg of cinnamon powder per dose, administered three times weekly for four consecutive weeks. The cinnamon was obtained from the Iranian Institute of Medicinal Plants (Tehran, Iran) and encapsulated to ensure standardized administration [40]. Supplementation was provided on training days, and participants were instructed to consume one capsule with a glass of water prior to each exercise session. To maximize compliance and ensure protocol fidelity, all supplement administrations were conducted under the direct supervision of the principal investigator. The selected dose and frequency were based on the study objective of evaluating the potential physiological effects of a practical, low-dose cinnamon supplementation regimen when combined with exercise training.

### 2.5. Outcome measurements

#### 2.5.1. Anthropometry and body composition

Anthropometric assessments were performed at baseline and immediately after the intervention under standardized measurement conditions. Participants were assessed barefoot and wearing light clothing. Body weight was obtained using a calibrated digital scale and recorded to the nearest 0.5 kg, while standing height was measured with a calibrated stadiometer to the nearest 0.1 cm. BMI was subsequently calculated as body weight in kilograms divided by height in meters squared (kg/m²) [46].

Chest and abdominal circumferences were assessed using a non-elastic, flexible measuring tape with a measurement resolution of 0.1 cm. Chest circumference was measured at the level of the xiphoid process, with participants standing upright, feet together, arms relaxed alongside the body, and breathing normally. The measurement was taken at the end of a normal expiration. Abdominal circumference was measured horizontally at the level of the umbilicus under the same standardized standing posture and respiratory conditions. Each circumference was measured three times, and the mean of the measurements was used for analysis; if repeated measurements differed by more than 1.0 cm, an additional measurement was obtained and the closest/mean values were recorded. All anthropometric measurements were performed by trained assessor, who followed a standardized measurement protocol throughout the study.

#### 2.5.2. Blood sampling

To assess changes in sex hormones, blood sampling was first performed from the participants' right arm vein. This sampling was performed in two stages. Once before the start of exercise training and supplement intake, and the second time 24 hours after the last training session. After 15 minutes of rest, the participants were placed in a sitting position and blood sampling was performed in a quiet environment. Blood samples were collected in a volume of 5 ml in special tubes containing coagulation gel. Then, the blood samples were transported to the laboratory at 4°C and the levels of estradiol, progesterone, FSH, and LH hormones were measured using special ELISA kits and according to standard protocols [47–49].

### 2.6. Statistical analysis

Descriptive statistics (mean ± standard deviation) were calculated for all study variables. The Kolmogorov–Smirnov test was employed to examine the normality of data distribution. In the study, a dependent t-test was used to compare pre-test and post-test values, and repeated measures analysis of variance (ANOVA) was used to examine within-group and between-group differences over time. All statistical analyzes were conducted using IBM SPSS Statistics, version 21.0 (IBM Corp., Armonk, NY, USA). A two-tailed significance level of *α* = 0.05 was considered, and *p*-values below this threshold were interpreted as statistically significant.

### 2.7. Ethics Approval and Consent to Participate

This study was conducted in full compliance with ethical principles outlined in the Declaration of Helsinki. Written informed consent was obtained from all participants, who were assured of the confidentiality of their data, as well as their voluntary right to participate and withdraw from the study at any time. The research protocol was reviewed and approved by the Research Ethics Committee of Bushehr University of Medical Sciences, Iran (Ethics Code: IR.BPUMS.REC.1403.319).

## 3. Results

### 3.1. Anthropometric characteristics and reproductive hormone profiles

Baseline characteristics were comparable between the E (age: 23.38 ± 1.80 years; height: 163.30 ± 3.49 cm) and E+S (age: 24.00 ± 2.48 years; height: 162.15 ± 5.03 cm) groups, with no significant between-group differences (all *p* > 0.05). After the 4-week intervention period, body weight and BMI decreased significantly in both groups (*p* = 0.001 for all within-group comparisons). Body weight decreased by 2.17% in the E group and 2.33% in the E+S group, while BMI declined by 2.18% and 2.26%, respectively. Chest circumference was significantly reduced in both groups (*p* = 0.001), with a greater percentage decrease in the E+S group (−1.73%) compared with the E group (−0.73%). Similarly, abdominal circumference decreased significantly in both groups (*p* ≤ 0.005), with larger reductions observed in the E+S group (−5.05%) than in the E group (−3.34%). However, none of the between-group differences in changes reached statistical significance (all *p* > 0.05) (Table 1).

**Figure 1.**
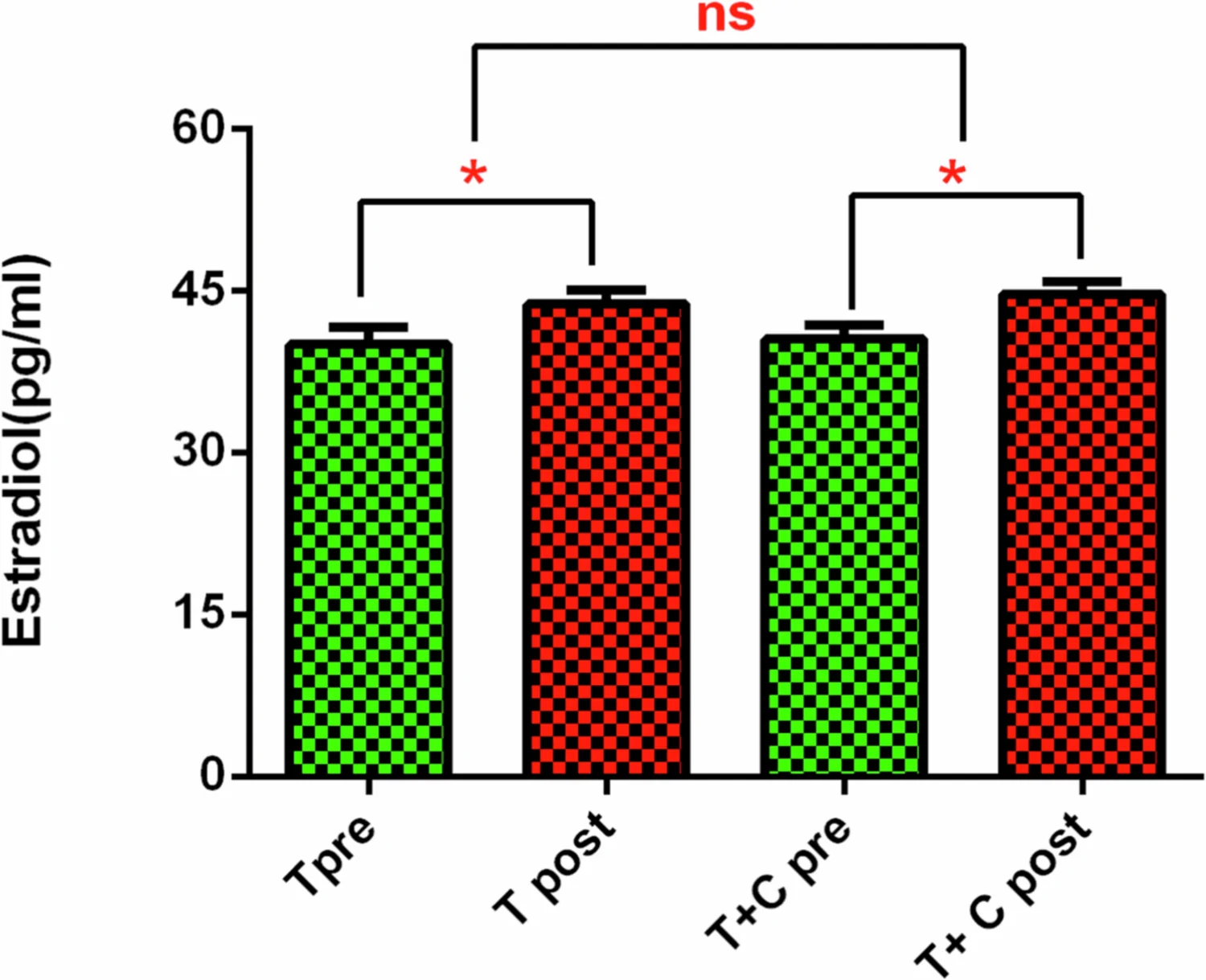
Changes in estradiol concentrations following exercise and exercise combined with supplementation. Data are presented as mean ± SD. Statistical analysis was performed using repeated-measures analysis of variance (ANOVA). T pre: exercise training pre-intervention; T post: exercise training post-intervention; T+C pre: exercise training + supplementation pre-intervention; T+C post: exercise training + supplementation post-intervention. Brackets indicate the statistical comparisons shown in the figure. Asterisks (*) indicate statistically significant differences (*p* < 0.05), whereas ns indicates a non-significant difference (*p* ≥ 0.05).

**Figure 2.**
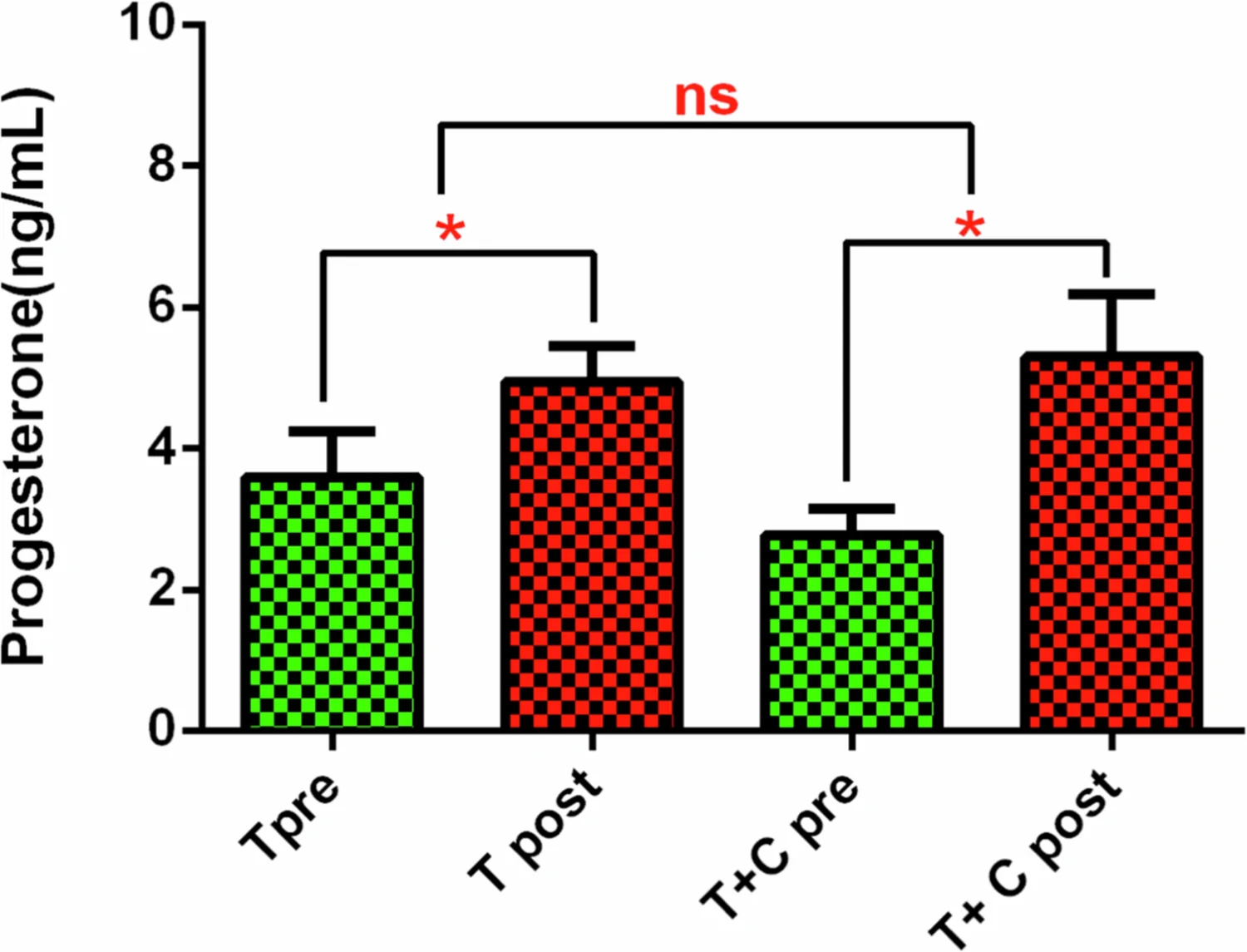
Changes in Progesterone concentrations following exercise and exercise combined with supplementation. Data are presented as mean ± SD. Statistical analysis was performed using repeated-measures analysis of variance (ANOVA). T pre: exercise training pre-intervention; T post: exercise training post-intervention; T+C pre: exercise training + supplementation pre-intervention; T+C post: exercise training + supplementation post-intervention. Brackets indicate the statistical comparisons shown in the figure. Asterisks (*) indicate statistically significant differences (*p* < 0.05), whereas ns indicates a non-significant difference (*p* ≥ 0.05).

**Figure 3.**
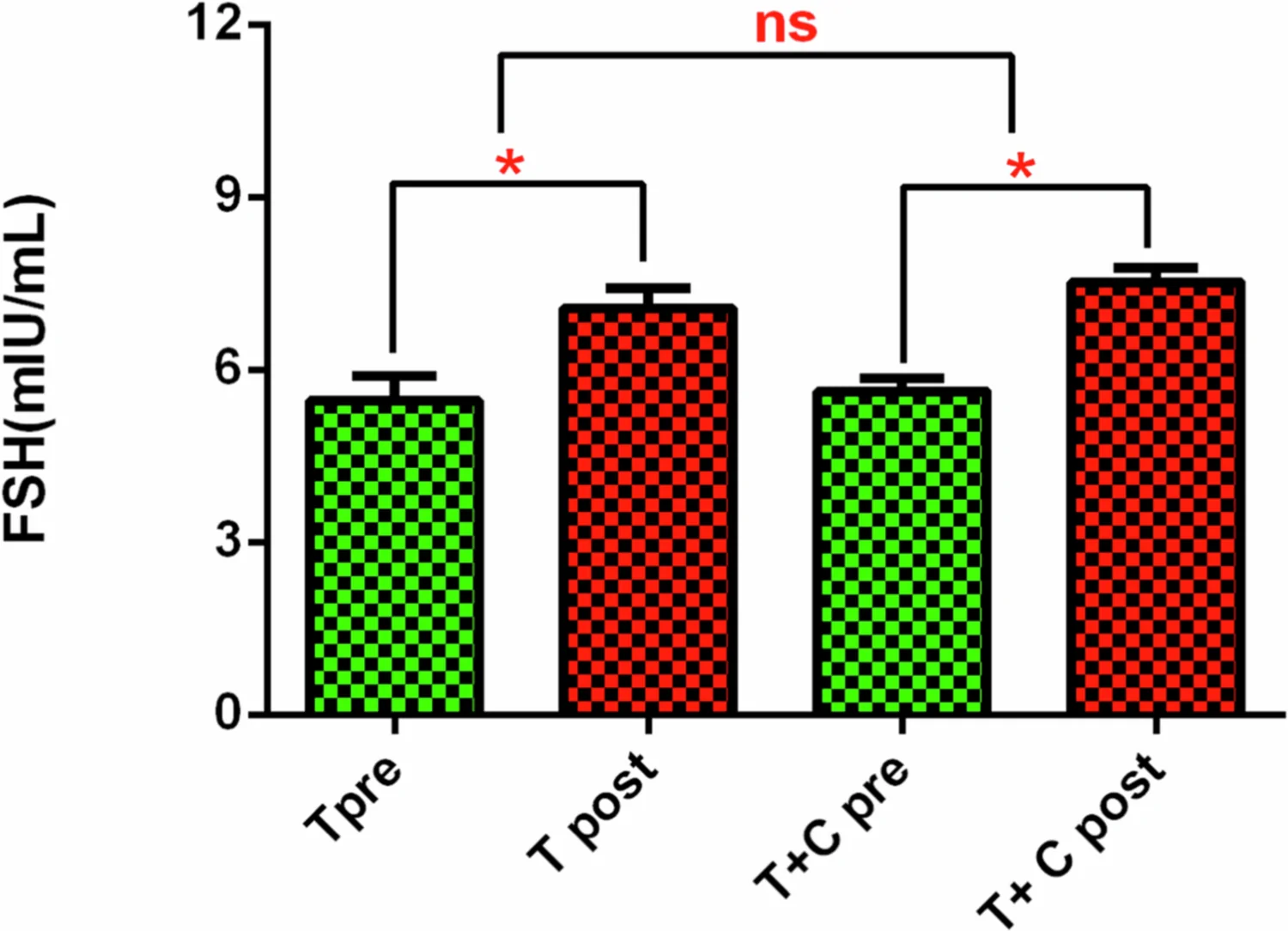
Changes in FSH hormone concentrations following exercise and exercise combined with supplementation. Data are presented as mean ± SD. Statistical analysis was performed using repeated-measures analysis of variance (ANOVA). T pre: exercise training pre-intervention; T post: exercise training post-intervention; T+C pre: exercise training + supplementation pre-intervention; T+C post: exercise training + supplementation post-intervention. Brackets indicate the statistical comparisons shown in the figure. Asterisks (*) indicate statistically significant differences (*p* < 0.05), whereas ns indicates a non-significant difference (*p* ≥ 0.05).

**Figure 4.**
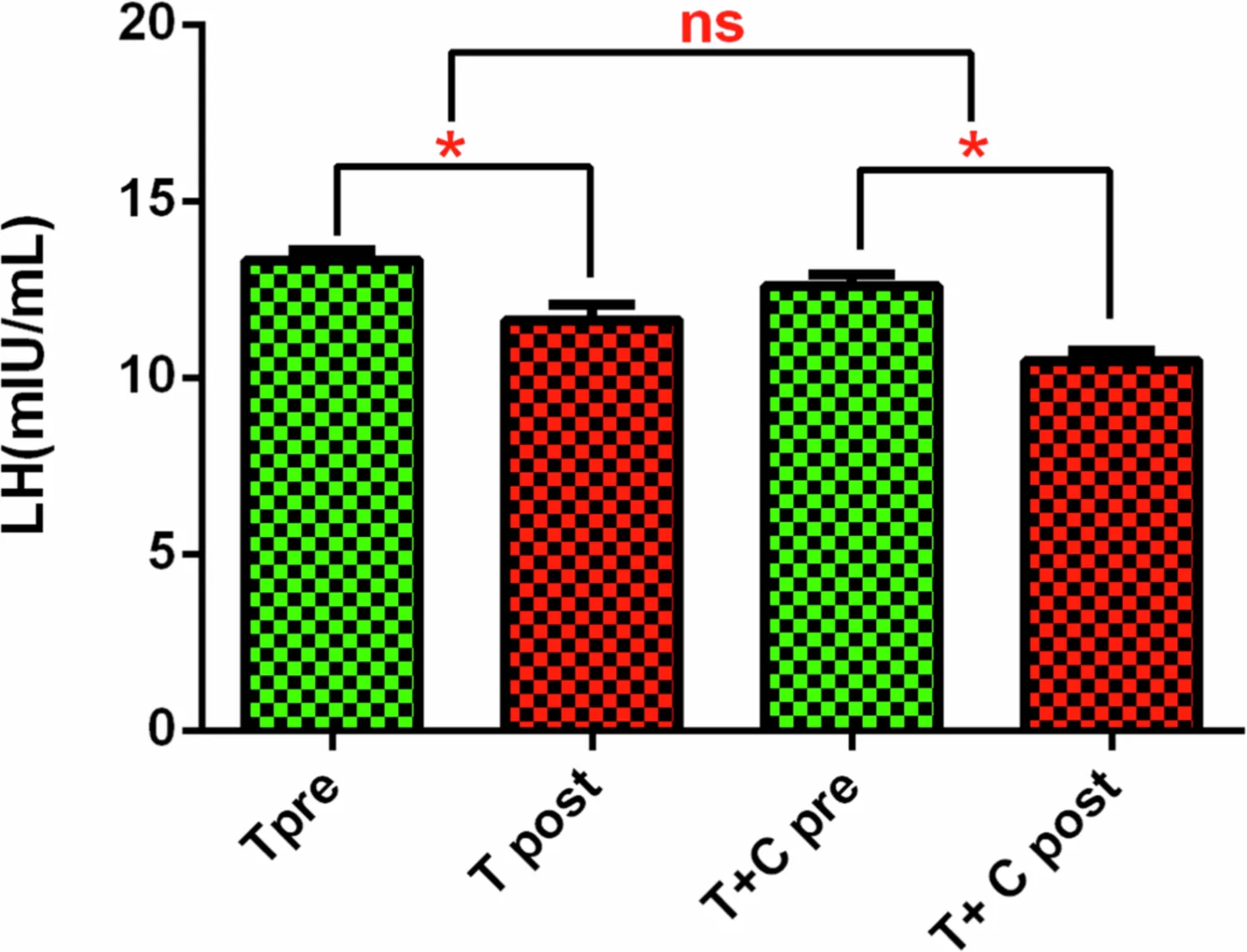
Changes in LH hormone concentrations following exercise and exercise combined with supplementation. Data are presented as mean ± SD. Statistical analysis was performed using repeated-measures analysis of variance (ANOVA). T pre: exercise training pre-intervention; T post: exercise training post-intervention; T+C pre: exercise training + supplementation pre-intervention; T+C post: exercise training + supplementation post-intervention. Brackets indicate the statistical comparisons shown in the figure. Asterisks (*) indicate statistically significant differences (*p* < 0.05), whereas ns indicates a non-significant difference (*p* ≥ 0.05).

Regarding reproductive hormonal, estradiol concentrations increased significantly in both groups (*p* = 0.001), with percentage increases of 9.40% and 15.26% in the E and E+S groups, respectively (Figure 1). Progesterone levels also increased significantly following the intervention, rising by 37.32% in the E group and 92.03% in the E+S group (*p* ≤ 0.005) (Figure 2). In addition, FSH concentrations increased significantly in both groups (*p* = 0.001), with percentage changes of 29.30% and 33.80% in the E and E+S groups, respectively (Figure 3). Conversely, LH concentrations decreased significantly in both groups, with reductions of 12.82% in the E group (*p* = 0.002) and 16.69% in the E+S group (*p* = 0.001) (Figure 4). Despite the different magnitudes of change observed between groups, none of the between-group comparisons was statistically significant for estradiol, progesterone, FSH, or LH (all *p* > 0.05) (Table 1).

**Table 1.** Changes in anthropometric characteristics and reproductive hormone profiles following four weeks of exercise alone or exercise combined with cinnamon supplementation in overweight and obese women.

Variable	Group	Pre-test	Post-test	% of changes	P_1_	P_2_
Body weight (kg)	E	73.15 ± 3.60	71.56 ± 3.53	−2.17	0.001*	0.589
	E+S	72.00 ± 6.69	70.32 ± 6.35	−2.33	0.001*	
Body mass index (kg/m^2^)	E	27.44 ± 1.34	26.84 ± 1.29	−2.18	0.001*	0.933
	E+S	27.37 ± 2.25	26.75 ± 2.27	−2.26	0.001*	
Chest circumference (cm)	E	97.38 ± 2.95	96.67 ± 3.10	−0.73	0.001*	0.226
	E+S	95.67 ± 3.63	94.01 ± 3.26	−1.73	0.001*	
Abdominal Circumference (cm)	E	97.15 ± 4.27	93.90 ± 5.86	−3.34	0.005*	0.707
	E+S	96.53 ± 3.43	91.65 ± 3.43	−5.05	0.002*	
Estradiol (pg/ml)	E	40.00 ± 5.84	43.76 ± 4.60	+9.40	0.001*	0.814
	E+S	40.49 ± 4.60	46.67 ± 4.15	+15.26	0.001*	
Progesterone (ng/mL)	E	3.59 ± 2.34	4.93 ± 1.81	+37.32	0.001*	0.286
	E+S	2.76 ± 1.37	5.30 ± 3.16	+92.03	0.005*	
FSH (mIU/mL)	E	5.46 ± 1.54	7.06 ± 1.31	+29.30	0.001*	0.116
	E+S	5.62 ± 0.86	7.52 ± 0.91	+33.80	0.001*	
LH (mIU/mL)	E	13.33 ± 1.05	11.62 ± 1.62	−12.82	0.002*	0.749
	E+S	12.58 ± 1.26	10.48 ± 0.95	−16.69	0.001*	

Data are expressed as mean and standard deviation; *: Indicates significant difference between means. E: Exercise; S: Supplement; *p*_1_-value: Pre-test and post-test comparison in group; *p*_2_-value: Baseline comparison between the two groups; % of changes: Percent change from pre-test to post-test [(post − pre)/pre × 100%].

### 3.2. Repeated-measures ANOVA assumptions and statistical model

A two-factor repeated-measures ANOVA was performed to evaluate the effects of time (pre- vs. post-intervention) and group (exercise vs. exercise plus cinnamon supplementation) on anthropometric and hormonal outcomes. Mauchly's test confirmed that the assumption of sphericity was satisfied (W = 1.00, χ^2^ = 0.00, *p* = 1.00).

#### 3.2.1. Anthropometric outcomes

Significant main effects of time were observed for all anthropometric variables, including body weight (F(1,24) = 146.14, *p* < 0.001, ηp^2^ = 0.86), BMI (F(1,24) = 171.42, *p* < 0.001, ηp^2^ = 0.88), chest circumference (F(1,24) = 60.97, *p* < 0.001, ηp^2^ = 0.72), and abdominal circumference (F(1,24) = 27.44, *p* < 0.001, ηp^2^ = 0.53), indicating significant reductions from baseline to post-intervention across both groups. No significant main effects of group were detected for body weight (*p* = 0.567), BMI (*p* = 0.918), chest circumference (*p* = 0.104), or abdominal circumference (*p* = 0.448). Likewise, no significant time × group interactions were observed for body weight (F(1,24) = 0.09, *p* = 0.768, ηp^2^ = 0.004), BMI (F(1,24) = 0.09, *p* = 0.768, ηp^2^ = 0.004), or abdominal circumference (F(1,24) = 1.10, *p* = 0.304, ηp^2^ = 0.04). However, a significant time × group interaction was identified for chest circumference (F(1,24) = 10.90, *p* = 0.003, ηp^2^ = 0.31), indicating that the magnitude of reduction differed between groups, with a greater decrease observed in the exercise plus supplementation group than in the exercise-only group (Table 2).

**Table 2.** Effects of exercise with and without cinnamon supplementation on anthropometric outcomes: repeated-measures ANOVA results.

Variable	Effect	F(1, 24)	*p*	ηp^2^
Body weight (kg)	Time	146.14	<0.001*	0.859
	Group	0.34	0.567	0.014
	Time × Group	0.09	0.768	0.004
Body mass index (kg/m^2^)	Time	171.42	<0.001*	0.877
	Group	0.01	0.918	0.000
	Time × Group	0.09	0.768	0.004
Chest circumference (cm)	Time	27.44	<0.001*	0.533
	Group	0.60	0.448	0.024
	Time × Group	1.10	0.304	0.044
Abdominal circumference (cm)	Time	60.97	<0.001*	0.718
	Group	2.85	0.104	0.106
	Time × Group	10.90	0.003*	0.312

M: Mean; SD: Standard Deviation; ηp^2^: Partial Eta squared.

#### 3.2.2. Reproductive hormone outcomes

Significant main effects of time were observed for all measured reproductive hormones, including FSH (F(1,24) = 65.69, *p* < 0.001, ηp^2^ = 0.73), LH (F(1,24) = 120.41, *p* < 0.001, ηp^2^ = 0.83), progesterone (F(1,24) = 24.68, *p* < 0.001, ηp^2^ = 0.51), and estradiol (F(1,24) = 51.54, *p* < 0.001, ηp^2^ = 0.68). Specifically, FSH, progesterone, and estradiol increased significantly over time, whereas LH decreased significantly following the intervention. No significant main effects of group were observed for FSH (*p* = 0.463), progesterone (*p* = 0.776), or estradiol (*p* = 0.704). Although LH demonstrated a borderline group effect (F(1,24) = 4.19, *p* = 0.052, ηp^2^ = 0.15), the difference did not reach statistical significance. Furthermore, no significant time × group interactions were identified for FSH (F(1,24) = 0.49, *p* = 0.490, ηp^2^ = 0.02), LH (F(1,24) = 1.27, *p* = 0.272, ηp^2^ = 0.05), progesterone (F(1,24) = 2.30, *p* = 0.142, ηp^2^ = 0.09), or estradiol (F(1,24) = 0.14, *p* = 0.711, ηp^2^ = 0.006), indicating comparable hormonal responses over time between the two intervention groups (Table 3).

**Table 3.** Effects of exercise with and without cinnamon supplementation on reproductive hormone profiles: repeated-measures ANOVA results.

Variable	Effect	F(1, 24)	*p*	ηp^2^
FSH (mIU/mL)	Time	65.692	<0.001*	0.732
	Group	0.557	0.463	0.023
	Time × Group	0.492	0.490	0.020
LH (mIU/mL)	Time	120.407	<0.001*	0.834
	Group	4.192	0.052	0.149
	Time × Group	1.265	0.272	0.050
Progesterone (ng/mL)	Time	24.682	<0.001*	0.507
	Group	0.083	0.776	0.003
	Time × Group	2.304	0.142	0.088
Estradiol (pg/ml)	Time	51.540	<0.001*	0.682
	Group	0.148	0.704	0.006
	Time × Group	0.141	0.711	0.006

M: Mean; SD: Standard Deviation; ηp^2^: Partial Eta squared.

## 4. Discussion

The present study aimed to investigate the effects of a selected exercise program combined with cinnamon supplementation on chest circumference, waist circumference, and reproductive hormones in overweight and obese inactive women. This study is among the first to examine the combined effects of targeted abdominal and chest exercises with cinnamon supplementation on both anthropometric and reproductive hormone outcomes in this population.

### 4.1. Body composition

The present findings indicate that both exercise training alone and exercise combined with cinnamon supplementation were effective in improving anthropometric outcomes, including reductions in body weight, BMI, and chest and abdominal circumferences in overweight and obese female students. Although the combined intervention group exhibited numerically greater reductions across most variables, a statistically significant between-group difference was observed only for chest circumference, where the exercise-plus-supplementation group demonstrated a greater reduction compared with the exercise-only group. For the remaining anthropometric variables, no significant group × time interactions were detected, indicating comparable efficacy between the two interventions.

Evidence on the combined effects of exercise and cinnamon supplementation on body composition is generally favorable but not uniform. Parseh et al. (2019) reported significant reductions in BMI and body fat percentage after six weeks of aerobic–resistance training with concurrent cinnamon intake in women with PCOS [50]. Similarly, Sabzevari Rad et al. (2025) demonstrated that eight weeks of Tabata training and cinnamon supplementation decreased body mass, BMI, and body fat percentage in all intervention groups, with the greatest fat loss in the training + cinnamon supplementation group in overweight and obese male soldiers [51]. Additionally, Delshad et al. (2023) also observed declines in body mass, BMI, and fat percentage after eight weeks of combined aerobic–TRX training with cinnamon supplementation in overweight inactive women [38]. In contrast, Hajimonfarednejad et al. (2018) found no significant anthropometric changes following 12 weeks of cinnamon supplementation alone in women with PCOS [42]. Evidence from exercise-only interventions also supports the beneficial effects of physical training on anthropometric outcomes. Atuegbu et al. (2014) reported that four weeks of moderate-to-vigorous submaximal bicycle exercise resulted in significant reductions in body weight and BMI among female university students [52].

The inconsistency across studies likely reflects heterogeneity in intervention design and study populations. Protocols varied substantially in duration (6–12 weeks), exercise modality and intensity, and the form/dose of cinnamon (powder vs. extract; with or without training). Moreover, participant characteristics differed with respect to BMI, hormonal milieu (e.g. PCOS status, concurrent hormonal therapy), and baseline metabolic health. Outcome selection also diverged, ranging from insulin resistance and adiposity indices to adipokines and molecular pathways. Collectively, these methodological and contextual differences provide a plausible explanation for the divergent findings and should be carefully considered when interpreting the literature.

Taken together, these findings suggest that intervention duration alone is unlikely to explain outcome differences; rather, the presence of an exercise stimulus appears to be decisive, with combined interventions producing more robust and consistent improvements. Mechanistically, repeated high-intensity or interval-based training induces cumulative adaptations which require several weeks to fully manifest [53–55]. Cinnamon may augment these adaptations by improving insulin sensitivity and facilitating substrate utilization [56,57]; however, in the absence of exercise, these metabolic effects may be insufficient to induce measurable changes in body composition, even over longer durations [42]. Therefore, both adequate intervention length and the synergistic interaction between exercise-induced and nutritionally mediated metabolic pathways are critical determinants of efficacy.

On the other hand, PCOS is a complex endocrine–metabolic disorder characterized by neuroendocrine dysregulation, ovarian dysfunction, and a high prevalence of metabolic abnormalities, particularly insulin resistance and compensatory hyperinsulinemia [58,59]. Unlike simple overweight/obesity, hyperinsulinemia in PCOS directly stimulates ovarian androgen production via insulin and IGF-1–mediated pathways, with IGF-1 further enhancing steroidogenesis through synergistic interactions with gonadotropins (FSH and LH) [60–63]. Increased IGF-1 bioavailability, partly due to reduced IGFBP-1 levels, amplifies these effects, disrupting folliculogenesis and contributing to anovulation and hyperandrogenism [64–68]. Consequently, insulin resistance in PCOS is a central driver of both metabolic and reproductive dysfunction [69]. In contrast, overweight or obese individuals without PCOS lack this heightened ovarian sensitivity to insulin/IGF-1 signaling; thus, interventions such as exercise and cinnamon supplementation primarily exert systemic metabolic effects rather than direct ovarian modulation. This fundamental mechanistic difference may underlie the divergent hormonal responses reported between PCOS and non-PCOS populations.

From a mechanistic perspective, the training paradigm employed in the present study closely resembles Tabata and high-intensity interval training, both of which are known to elicit robust metabolic adaptations. Such protocols enhance post-exercise energy expenditure and fatty acid oxidation, partly mediated by elevations in catecholamines and growth hormone, as well as activation of the AMPK–PGC-1α signaling axis that promotes mitochondrial biogenesis. These adaptations collectively contribute to improved body composition, supporting evidence that short-duration, high-intensity exercise is an efficient strategy for reducing adiposity [53–55]. In addition, this type of exercise has been shown to promote mitochondrial biogenesis in skeletal muscle, thereby enhancing cellular metabolic efficiency and overall metabolic health. It also induces a prolonged post-exercise metabolic perturbation characterized by elevated enzymatic activity and increased substrate turnover. Post-exercise physiological processes such as lactate and H⁺ clearance, glycogen resynthesis, and sustained elevations in growth hormone further contribute to enhanced lipid oxidation, thereby facilitating fat reduction and weight loss [70,71]. Moreover, improvements in glucose metabolism induced by this training modality, together with the substantial energy expenditure during and after exercise, may act as a potent metabolic catalyst for adipose tissue reduction [72]. Consistent with previous literature, these integrated adaptations are associated with improvements in body composition parameters, including body mass index, fat mass (percentage and absolute), and waist circumference [73], particularly in women [71]. In parallel, exercise—particularly interval-based modalities such as walking and cycling, or combined formats including circuit and boot camp training—has been associated with favorable metabolic and reproductive outcomes in women with obesity, although heterogeneity in exercise prescriptions and outcome measures remains considerable [74].

Cinnamon supplementation may further potentiate these effects through complementary metabolic pathways. Experimental and clinical evidence suggests that cinnamon can modulate body fat, particularly abdominal adiposity, via mechanisms involving reduced intestinal lipid absorption and enhanced fatty acid oxidation [75,76]. In addition, bioactive compounds such as hydroxychalcone exert insulin-mimetic effects, improving insulin sensitivity and facilitating glucose uptake, while methyl hydroxychalcone has been implicated in promoting cellular uptake of glucose and free fatty acids [56,57]. Given the central role of insulin in lipid metabolism, these properties may augment fat utilization and contribute to improvements in body composition when combined with exercise interventions.

### 4.2. Sex hormones

The results further suggest that both interventions induced favorable changes in reproductive hormones, characterized by significant increases in estradiol, progesterone, and FSH, alongside a significant reduction in LH. While the exercise-plus-cinnamon group generally showed larger magnitude changes compared with the exercise-only group, these differences did not translate into statistically significant group × time interactions. This indicates that, despite consistent within-group improvements, the addition of cinnamon supplementation did not produce a superior hormonal response beyond that achieved by exercise training alone.

Evidence from exercise-based interventions conducted in female populations suggests that reproductive hormones may respond favorably to structured training programs, although the magnitude and direction of these responses vary across studies. Atuegbu et al. (2014) reported that four weeks of moderate-to-vigorous submaximal bicycle exercise in female university students resulted in significant reductions in estrogen, FSH, and LH concentrations, accompanied by a significant increase in progesterone levels [52]. Similarly, Ramadan et al. (2025) observed that ten weeks of either high-intensity interval training or traditional resistance training in young women significantly increased estrogen concentrations and reduced FSH levels, while LH remained unchanged [77]. Although the hormonal patterns reported in these studies are not identical to those observed in the present investigation, they collectively support the notion that exercise training can modulate reproductive endocrine function in women.

The variability among studies may be explained by differences in participant characteristics, exercise prescription, intervention duration, and hormonal assessment protocols. Factors such as age, adiposity, baseline fitness level, menstrual-cycle phase, and energy balance are known to influence endocrine responses to exercise and may contribute to the heterogeneous findings reported across female populations.

Direct evidence regarding the effects of cinnamon supplementation on reproductive hormones in overweight or obese women without PCOS remains limited. Therefore, interpretation of the potential endocrine role of cinnamon should be made cautiously. Previous investigations conducted in distinct populations, including trained male athletes and women with PCOS, have reported inconsistent hormonal responses to cinnamon supplementation [40,42,78]. Because these populations differ substantially from the participants included in the present study with respect to sex, training status, metabolic characteristics, and endocrine physiology, such findings should be considered indirect evidence and primarily serve to provide mechanistic context rather than direct support for the current results.

In women with PCOS, chronic insulin resistance, hyperinsulinemia, and altered ovarian sensitivity to insulin and IGF-1 signaling contribute to reproductive endocrine dysfunction and may influence responsiveness to nutritional interventions [58,59]. Likewise, hormonal adaptations observed in highly trained athletes may reflect physiological conditions that differ considerably from those of sedentary overweight or obese women [78]. Consequently, extrapolation of findings from these populations should be undertaken with caution.

Taken together, the current evidence suggests that exercise training is likely the principal driver of the hormonal adaptations observed in the present study. Although cinnamon supplementation may influence endocrine regulation through mechanisms related to insulin sensitivity, oxidative stress, and metabolic control, the absence of significant group × time interactions for reproductive hormones indicates that any additional effect of cinnamon beyond exercise alone remains uncertain under the conditions examined in this study.

Previous studies have suggested that cinnamon may influence endocrine and reproductive pathways and LH and FSH levels through modulation of insulin sensitivity, oxidative stress, and hypothalamic–pituitary–gonadal (HPG) axis activity. However, the absence of significant group × time interactions for reproductive hormones in the present study indicates that any additional endocrine effect of cinnamon beyond exercise remains uncertain and warrants further investigation [79]. Given the direct relationship between insulin resistance and elevated LH and testosterone levels, the insulin-sensitizing properties of cinnamon may contribute to a reduction in LH levels [80]. Furthermore, cinnamaldehyde, a key compound in cinnamon, has been shown to enhance LH secretion by stimulating nitric oxide synthesis [81]. This compound also promotes norepinephrine release through calcium ion binding to the cell membrane and cyclic AMP activation [82]. Additionally, leptin has been reported to increase FSH secretion via the activation of neuronal nitric oxide [83]. Cinnamon also exhibits notable antioxidant properties due to the presence of polyphenol A [84]. This antioxidant potential may play a protective role by reducing free radicals and reactive oxygen species (ROS), which are known to impair reproductive function and increase during intense physical activity.

Exercise appears to modulate reproductive and steroid hormone regulation primarily through its influence on the HPG axis, which is closely integrated with autonomic and hypothalamic–pituitary–adrenal (HPA) activity. In response to physical stress, these neuroendocrine systems coordinate steroidogenic signaling, whereby exercise intensity and physiological load determine the direction of hormonal output [85]. When the exercise stimulus remains within a tolerable range, steroidogenic pathways tend to favor anabolic and reproductive hormone availability, whereas excessive or maladaptive stress may shift endocrine output toward catabolic glucocorticoid dominance, thereby altering gonadal hormone balance [85].

Within this regulatory framework, exercise-induced alterations in gonadotropin signaling may contribute to downstream modulation of ovarian steroidogenesis, including estrogen and estradiol production. Variations in gonadotropin pulsatility and receptor sensitivity are considered key mediators of these adaptations, influencing ovarian responsiveness and circulating sex steroid concentrations [86]. Additionally, exercise-related changes in systemic inflammatory mediators and metabolic status may indirectly affect hypothalamic regulation of gonadotropin release, further shaping reproductive endocrine responses [85,87]. Collectively, these integrated neuroendocrine and metabolic interactions provide a plausible mechanistic basis for the observed exercise-induced adaptations in FSH, LH, estrogen, and estradiol in women, highlighting the sensitivity of the reproductive axis to physiological training stress [87–89].

Direct comparisons between the exercise-only group and the exercise-plus-supplementation group revealed no statistically significant differences in the changes of FSH, LH, estradiol, progesterone, or abdominal circumference. These findings suggest that the observed improvements in these outcomes were primarily driven by time or training effects rather than the addition of cinnamon supplementation. Various reasons such as the training period, supplement dosage, duration of supplement use, and lack of accurate recording of food intake could have caused the lack of difference between the two groups, which are presented in the study limitations section.

The observed increases in estradiol, progesterone, and FSH, together with a reduction in LH, indicate significant pre-to-post changes in reproductive hormone concentrations following the study period. However, these changes should be interpreted cautiously because menstrual-cycle phase was determined based on self-report, and pre- and post-intervention blood samples were not necessarily collected at the same physiological stage of the menstrual cycle [79]. From a reproductive standpoint, this hormonal profile is generally associated with improved fertility potential. Elevated estradiol supports follicular growth and endometrial proliferation, while increased progesterone reflects enhanced luteal function, both of which are essential for successful implantation and early pregnancy maintenance [90]. The rise in FSH may facilitate follicular recruitment and maturation [91], whereas the reduction in LH—particularly relevant in conditions such as PCOS, where LH hypersecretion is common—may reflect a normalization of gonadotropin pulsatility and a more balanced LH/FSH ratio, which is critical for ovulatory function [92,93].

Although the observed within-group hormonal changes may reflect beneficial endocrine adaptations to exercise training, the absence of statistically significant group × time interactions indicates that the additional effect of cinnamon supplementation beyond exercise alone remains unconfirmed. This lack of differential effect may be attributable to methodological constraints such as limited sample size, inter-individual variability in hormonal responsiveness, and the relatively short duration of the intervention. Accordingly, while the pattern of changes is suggestive of potential improvements in reproductive endocrine function, these findings should be interpreted with caution, and further well-powered, longer-duration randomized controlled trials are warranted to clarify the potential additive role of cinnamon supplementation.

### 4.3. Strengths and limitations

A major strength of this study lies in its novel dual-intervention design, which combined exercise with cinnamon supplementation to evaluate their synergistic impact on anthropometric and hormonal parameters in overweight and obese women. The randomized allocation of participants into exercise-only and exercise + supplement groups, along with the controlled setting of the intervention, further strengthens the internal validity of the findings.

Nevertheless, several limitations of the present study should be clearly acknowledged. First, the absence of a true control group (i.e. no exercise/no supplement) limits the ability to isolate the independent effects of the interventions from natural variation over time. Second, the relatively short duration of the intervention (four weeks) may not have been sufficient to elicit stable or clinically meaningful adaptations in endocrine function, particularly for outcomes such as sex hormone regulation and fertility-related parameters that often require longer exposure to manifest. It is plausible that a longer intervention period would produce more pronounced and sustained hormonal and physiological responses. Third, the small sample size reduces the statistical power and generalizability of the findings. Additionally, dietary intake—a critical factor influencing hormonal balance—was neither controlled nor systematically recorded, which may have introduced variability in the results. Furthermore, due to logistical and resource limitations, exercise intensity was not objectively monitored or quantified throughout the intervention period. As exercise intensity is an important determinant of physiological and endocrine adaptations, the absence of this information limits the interpretation of the dose–response relationship between exercise and the observed outcomes. Future studies are encouraged to assess and report exercise intensity using objective measures such as heart rate monitoring, ratings of perceived exertion, or workload-based indices.

Furthermore, fertility was not directly assessed through clinical markers such as ovulation, pregnancy, or oocyte quality, which limits the extrapolation of results to reproductive outcomes. Another limitation is that luteal phase status was determined based on self-report rather than objective hormonal or ovulatory markers, which may have introduced misclassification bias. In addition, menstrual cycle phase was not objectively confirmed using validated methods such as ovulation testing, basal body temperature monitoring, or hormonal assessment. Given the natural variability in menstrual cycle length among participants, blood samples may not have been collected at identical physiological stages of the luteal phase before and after the intervention. This variability could have influenced circulating concentrations of estradiol, progesterone, FSH, and LH, thereby limiting the precision and interpretability of hormone-related findings. Therefore, the hormonal outcomes reported in the present study should be interpreted with caution.

Moreover, due to logistical and resource constraints, key androgenic markers such as testosterone were not assessed, despite their recognized importance in evaluating reproductive and endocrine function, particularly in populations with conditions such as PCOS. The omission of testosterone limits the comprehensiveness of the hormonal profile and the ability to fully interpret endocrine adaptations. Future studies should objectively verify menstrual cycle phase using validated approaches, including ovulation predictor kits, basal body temperature monitoring, or hormonal confirmation, and should standardize blood sampling according to specific menstrual cycle days. Such procedures would improve the reliability of endocrine assessments and allow for more accurate evaluation of intervention-induced hormonal changes. In addition, future investigations should objectively monitor exercise intensity to better characterize the training stimulus and determine its contribution to anthropometric and hormonal adaptations. Future studies are therefore encouraged to include a broader panel of sex hormones, including total and free testosterone, alongside longer intervention durations, larger sample sizes, comprehensive dietary monitoring, and true control and placebo groups to provide a more complete understanding of the underlying mechanisms and clinical relevance of such interventions.

## 5. Conclusion

The present findings highlight the potential value of a practical, easily implementable exercise program for overweight and obese women, with both study groups demonstrating favorable changes in body composition, including reductions in abdominal and chest circumferences, and reproductive hormone concentrations. It suggests that incorporating accessible exercise-based strategies may be relevant to improving anthropometric and hormonal profiles in this population. However, because no non-exercise control group was included and menstrual-cycle phase was not objectively standardized, these hormonal changes cannot be attributed specifically to exercise or interpreted as evidence of improved reproductive function or fertility. The addition of cinnamon did not produce significant between-group differences for abdominal circumference or reproductive hormones. Given the small sample size, limited dietary control, and uncontrolled habitual physical activity, these findings should be considered preliminary. Larger, adequately controlled trials with longer follow-up and standardized assessment of menstrual-cycle phase are warranted to establish the reproducibility and clinical relevance of these observations.

## Data Availability

The datasets used and/or analyzed during the current study are available from the corresponding author upon reasonable request.
